# New Insights into the Genetic Regulation of Plasmodium Falciparum Obtained by Bayesian Modeling

**Published:** 2007-11-29

**Authors:** Svetlana Bulashevska, Ezekiel Adebiyi, Benedikt Brors, Roland Eils

**Affiliations:** 1 Division ‘Theoretical Bioinformatics’, German Cancer Research Center (DKFZ), Im Neuenheimer Feld 280, 69120 Heidelberg, Germany; 2 Department of Computer and Information Sciences, Covenant University, PMB 1023, Ota, Nigeria; 3 Department ‘Bioinformatics and Functional Genomics’, Institute of Pharmacy and Molecular Biotechnology (IPMB), University of Heidelberg, Germany

## Abstract

The most fatal and prevalent form of malaria is caused by the bloodborne pathogen *Plasmodium falciparum* (henceforth P.f). Annually, approximately three million people died of malaria. Despite P.f devastivating effect globally, the vast majority of its proteins have not been characterized experimentally. In this work, we provide computational insight that explore the modalities of the regulation for some important group of genes of P.f, namely components of the glycolytic pathway, and those involved in apicoplast metabolism. Glycolysis is a crucial pathway in the maintenance of the parasite while the recently discovered apicoplast contains a range of metabolic pathways and housekeeping processes that differ radically to those of the host, which makes it ideal for drug therapy.

We have been able to validate some of our findings from available literature and therefore provide a basis to give theoretical insight for some genes regulations, which has not been characterized experimentally.

## 1 Introduction

The most fatal and prevalent form of malaria is caused by the bloodborne pathogen *Plasmodium falciparum* (henceforth P.f). Annually, approximately three million people died of malaria. Also, hundreds of millions of people in a year become clinically ill. The negative influence of these results is huge and its socioeconomic impact is beyond measure. This influence is particularly prominent in the Africa continent, where an estimated US$12 billion is been lost yearly (2; 9). Reports has shown that the parasites is growing resistance to existing drugs. Therefore, there is a huge and urgent need to discover and validate new drug or vaccine targets to enable the development of new treatments for malaria (4). The ability to discover these drug or vaccine targets can only be enhanced from our understanding of the detailed conceptual view of the gene regulatory circuitry of P.f.

Previously, two computational studies have attempted to characterize the genetic regulation of glycolytic genes and those specific for the apicoplast, namely, Khanin and Wit, 2004 and Barrera et al. 2004. Their models could only show whether two genes interacted but were unable to explain the modalities of the genes regulation. In this work, we provide computational insight into the regulation of *Plasmodium falciparum* genes in glycolysis and apicoplast pathways using the time-series gene expression measurements of the intraerythrocytic development cycle (dataset of Bozdech et al. (5)). Our work can be said to be the first study that looks closely at important group of genes in P.f and attempt to elucidate the genes regulatory connections. The dataset of (5) has been shown (25) to compare very well with the other microarray dataset of (16), a recent comparison of this dataset to the results on other strains of P.f can be found in (18). And since the two existing computational genetic networks for P.f were induced using Bozdech et al. results, we made this data our basic dataset in this study. For the reconstruction of genetic regulatory interactions from the data, we applied Bayesian inference of the probabilistic model, previously developed and tested on the yeast *S. cerevisiae* (3). This model is based on the biologically motivated Boolean logic semantics, but a probabilistic one i.e. giving the possibility to incorporate uncertainty about the noisy data and the noisy process of genetic regulation. The framework allows for a particular gene to find a set of its regulators given a particular Boolean logic function governing this regulation. Boolean logic is a simple and particularly suitable way to model the working principles of the *cis-*regulatory elements. The ‘OR’ logic represents that a gene can be activated by one of a few different possible transcription factors. In case of ‘OR-NOR’ logic, a gene is regulated by a set of possible activators and a set of possible inhibitors. The gene is transcribed if and only if one of its possible activators is active and it is not repressed by one of its possible repressors. We employ this kind of probabilistic graphical models with Boolean logic semantics for modeling the genetic regulatory interactions of P.f.

The write-up is organized as follows. In Section 2, we describe the systems and methods applied in our modeling and analysis of the pathway and metabolism under consideration. Section 3 contains our results and discussion of the implication of these results. We end this paper in Section 4 with a conclusion and further-work.

## 2 Systems and Methods

### 2.1 The model of gene regulatory interactions

**Figure 1:**
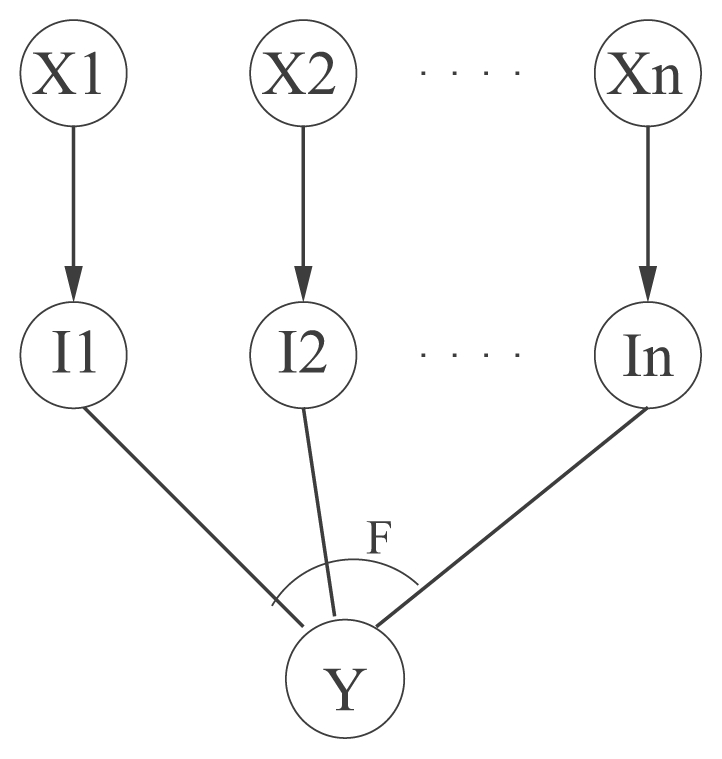
Model of gene regulatory interactions, F—Boolean function ‘OR’.

**Figure 2:**
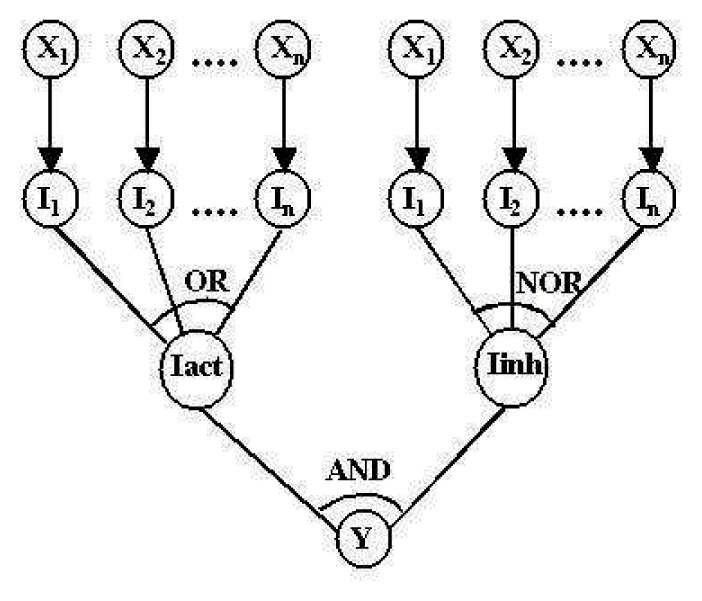
Complex model of gene regulatory interactions with activators and inhibitors (“OR-NOR” regulation).

Here, we describe the probabilistic graphical model underlying our approach of inferring gene regulatory interactions of P.f. We assume that genes have only two states—active and not active, and model them with stochastic variables having binary values. The general structure of the gene interaction in our models is represented by a directed graph (see Fig. 1). We assume that the variable *X_i_* (regulator) can execute its influence on the variable Y (regulatee) independently of other possible regulators *X*_1_, …, *X_n_* of Y. The biological mechanism underlying this modeling assumption is the binding of protein transcribed by the regulator to the DNA of the regulatee. This process is not deterministic, rather each gene *X_i_* can regulate the gene Y with probability *θ_i_* and can fail to do this with probability 1 − *θ_i_*. In the graphical model intermediate variables *I*_1_, …, *I_n_* are introduced, through which the variables *X*_1_, …, *X_n_* execute their influence on a given common effect variable Y. Each intermediate variable *I_i_* has only one parent, the variable *X_i_*. It’s probability distribution is defined as follows: given that *X_i_* = 1, *I_i_* takes the value 1 with probability *θ_i_* and the value 0 with probability 1 − *θ_i_*, respectively. Given that *X_i_* = 0, *I_i_* takes the value 0 with probability 1. The combined regulatory influence on the variable *Y* is calculated as the boolean function *F* on the input variables *I*_1_, …, *I_n_*. If *X*_1_, …, *X_n_* are activators, then the state of the variable *Y* is *F*(*I*_1_,…, *I_n_*); if *X*_1_, …, *X_n_* are inhibitors, the state of *Y* is 1 − *F*(*I*_1_, …, *I_n_*). The boolean “interaction function” F defines in which way the intermediate effects *I_i_*, and indirectly the variables *X_i_*, interact. Here, we consider the interaction function ‘OR’. The semantics of the ‘OR’-function implies that the variables *X_i_* are each assumed to be sufficient to influence Y. In the present work we apply two models: the simple ‘OR’-model with activatory regulation and the complex ‘OR-NOR’-model with activatory and inhibitory regulation. In the complex model, the regulatory influences of multiple activators and multiple inhibitors are combined with ‘AND’-function as depicted in Figure 2.

Introduction of the hidden state variables *I_i_* allows to insert “noise” into the Boolean logic based models. It allows to model that the biological mechanism of the regulation of one gene by another could be inhibited for unknown reasons. Thus, the input variables can be considered as observables from which we make our noisy measurements, while the hidden variables have the “true” latent biological values.

### 2.2 Bayesian model selection

We employ the Bayesian methodology for learning the structure and parameters of the model from data. The Bayesian approach addresses the problem as calculating the posterior probability of a model given data for a collection of candidate models and selecting the most probable model. Suppose that the data *D* has been generated by a model *m*, one of a set *M* of candidate models, *m* ∈ *M*. If *p*(*m*) is the prior probability of model *m*, then the posterior model probability by Bayes rule is *p*(*m|D*) ∝ *p*(*D*|*m*)*p*(*m*). The marginal likelihood *p*(*D*|*m*) = ∫ *p*(*D*|m, *θm*)*p*(*θ_m_*|*m*)*dθ_m_*, where *p*(*θ_m_*|*m*) is the prior distribution of model parameters *θ_m_* for model *m*. The calculation of the marginal likelihood is the general computational bottleneck of the Bayesian methodology, since the integral is analytically tractable only in certain restricted examples, namely, when a prior distribution for the parameters of the model exists, so that the integral will have a closed form solution (*conjugate prior*). The models considered here are intractable (3).

Markov Chain Monte Carlo (MCMC) stochastic simulation techniques facilitate the Bayesian inference. MCMC generates samples from the joint posterior distribution *p*(*m,θ_m_*|*D*) allowing to estimate the posterior parameter probability *p*(*m,θ_m_*|*D*). One of the MCMC approaches is Gibbs sampling. Gibbs sampling reduces the problem of dealing simultaneously with a large number of unknown parameters in a joint distribution into a simpler problem of dealing with one variable at a time, iteratively sampling each from its full conditional distribution given the current values of all other variables in the model.

The software OpenBUGS (BUGS stands for Bayesian Updating with Gibbs Sampling) is the general purpose software for Gibbs sampling on graphical models (23). OpenBUGS provides a declarative language for specifying a graphical model, i.e. the specification of the model likelihood and of the prior distributions for all parameters is required. The output of Markov chain simulation is used to summarize the posterior distributions of the variables of interest. The present approach utilizes the Linux version of the software OpenBUGS.

Our problem of model selection is formulated as follows: given the data on the gene *Y* and its potential regulators *X*_1_, …, *X_p_*, for a given boolean logic function *F*, identify the subset *X*_1_, …, *X_n_* of actual regulators of *Y*. We substitute the model indicator *m* ∈ *M* with the *variable indicator* γ = (γ_1_, …, γ*_p_*), a binary vector, representing which of the *X*_j_, *j* = 1, …, *p* should be included in the desirable “true” model. This allows the consideration of one joint space of the model parameters and the variable indicator, keeping the dimensionality constant across all possible models. By introducing the variable indicator, the “OR” model may be written as:

$$Y∼Bernoulli\left(1-\prod_{i=1}^{n}{\left(1-\theta_{i}\right)}^{\gamma_{i}X_{i}}\right),$$

where *Y* and *X_j_* is the observed data and *θ* and γ are the parameters. This represents the specification of the model likelihood.

The Bayesian approach requires specification of prior distributions for the model parameters. We defined the priors for the parameters *θ_j_* with Beta distribution, since Beta distribution constrains the parameters to the [0,1]-interval. We use a hierarchical formulation of the distribution, i.e. with hyperparameters *a_j_* and *b_j_*:

$$\theta_{j}∼Beta(a_{j},b_{j})$$

The hyperparameters *a*_j_ and *b_j_* are defined in two different ways, dependently on the parameters γ*_j_*. If γ*_j_* = 1, they are defined equal to 1, therefore making the prior non-informative (*Beta*(1,1)). In the case γ*_j_* = 0, the parameters are called (*pseudopriors*). The pseudopriors may be chosen in a way to help increasing the efficiency of the sampling procedure. Efficient performance can be achieved when the moves of the MCMC chain between different models γ could be “local”. Therefore, the so called *proposal* densities for the pseudopriors can be used, which are being estimated by a *pilot run* of the MCMC for the *saturated* model, i.e. the model where all terms γ*_j_* = 1 for all *j*. Thus, we calculate the hyperparameters *a_j_* and *b_j_* by the formulas (*method of moments*):

$$\begin{array}{l} a_{j}+b_{j}=\frac{mean_{j}(1-mean_{j})}{var_{j}}-1,\\ a_{j}=(a_{j}+b_{j})mean_{j},\\ b_{j}=(a_{j}+b_{j})(1-mean_{j}), \end{array}$$

where *mean_j_* and *var_j_*, the mean and the variance of the parameters *θ_j_*, are estimated from the pilot run of the saturated model.

Next, one must define the prior distribution for the variable indicator γ. Since the terms γ*_j_* are independent, the prior can be decomposed into independent Bernoulli distributions for each term: γ*_j_* ∼ *Bernoulli*(π*_j_*), where π*_j_* is the prior probability to include term *j* into the model. The prior π*_j_* = 0.5 is non-informative in the sense of favoring all models equally, but is not non-informative with respect to the model size. To favor more parsimonious models (i.e. with small number of actual regulators), we used π*_j_* = 0.2.

The runs of the MCMC were summarized and monitored for convergence using R-package CODA (http://cran.r-project.org).

We obtained the Markov chain samples on the parameters γ*_j_* and *θ_j_*. For the ‘OR’-model, we used 20000 iterations of the Markov chain for the burn-in time and 50000 iterations for the parameter estimations. For the complex ‘OR-NOR’-model, we used 30000 iterations for the burn-in, and 100000 iterations for estimations. If the frequency of 1s in the chain for γ*_j_* exceeded 0.7, we assumed that γ*_j_* = 1and the respective regulator should be included in the “true” model. Otherwise, the regulator *j* should be excluded.

We apply learning the ‘OR’ and ‘OR-NOR’ models from data for each gene, considering all other genes in the dataset as candidate regulators. Hence, in our approach, bidirectional regulations might be inferred i.e. the gene *X* might be deduced to regulate *Y*, and the gene *Y* might be deduced to regulate *X*. Thus, cycles can be present in the global picture of genes regulation. This is opposite to other approaches based on graphical models such as e.g. Bayesian networks, where the learning of all interactions at once, using global criteria, is executed. The “local” model learning, presented in this paper, is of greater advantage, since it is capable of capturing the biological reality (feedback regulation etc.) more adequately.

### 2.3 Model checking

After the execution of the MCMC sampling and the estimation of the variable indicator γ, the check of goodness-of-fit of the model to data is required, to check whether the model assumptions were appropriate. Bayesian model checking uses the *posterior predictive distributions* (10). The goal is to perform posterior predictions under the model and to assess the discrepancy between predicted and observed data. If the model is reasonably accurate, the predicted data should be similar to the observed data.

Here, we wish to check the ability of the concrete regulatory model, defined by the inferred vector γ, to predict the state of the gene *Y* from the states of its regulators.

In the present framework, the posterior predictions can be computed by simulation: in every MCMC loop, a replicate *y^rep^* is generated conditionally on the currently generated parameters *θ*. (Note that here fixed binary values for γ, estimated previously by the model learning, are used.) Based on the MCMC simulations, the estimate *E*(*y^rep^*) of the replicate can be made. The replicate is generated for each *i* = 1, …, *N*, where *N* is the number of samples in the *Y* dataset. Then, the individual observations of *Y* in the dataset *y_i_*, *i* = 1, …, *N* must be compared to the replicate data. For the comparison, we use the residual function *r_i_* = |*y_i_* −, *E*(*y_i_^rep^*)|. Observations, for which the residual is not close to 0, indicate some lack-of-fit of the model and should be regarded as outlier. We regarded the residual as not close to 0, if its absolute value exceeded one estimated *var*(*y_i_^rep^*). We calculate the model prediction accuracy as the percentage of non-outliers.

### 2.4 Types of regulatory situations considered

Our data is the time-series data, i.e. we have measurements of genes at subsequent time points *t* = 1, …, *T*, where *T* = 53. The true biological time resolution of the gene transcription and activation is yet unknown. It can be assumed, that a gene is active at the same time point as its activators, or, that it becomes active at the next time point. We refer to the first situation as ‘simultaneous’ regulation, and to the second as ‘time delay’ regulation. Both situations are considered in the present paper.

We treat gene measurements at each time point as statistical data samples. In the case of ‘simultaneous’ regulation, the state of a gene in the sample *t* depends on the states of its regulators in the same sample. Here, we have 53 data samples. In the case of ‘time delay’ regulation, the state of a gene in the sample *t* depends on the states of its regulators in the sample *t* − 1, so we have 52 data samples.

### 2.5 Data discretization

For the discretization of the continuous gene expression values into two states (0 - not active, 1 - active) we used a vector quantization technique based on the clustering algorithm k-means. For each gene we clustered its expression values into two groups by the k-means algorithm with two initial values: 0 and the maximum expression value of the gene.

## 3 Results and Discussion

### 3.1 Glycolysis pathway

From the public database PlasmoDB (http://plasmodb.org), we harvested twenty genes that are known to be involved in the glycolysis pathway. The hypothetical functions for each of these genes are presented in the Table 1.

We found the time-resolved gene expression profiles of the eighteen of these genes in the dataset of (5). We averaged the values from multiple oligonucleotides representing same gene. Then we discretize the continuous gene expression values into binary values 1 and 0 representing that the gene is active and not active.

**Figure 3:**
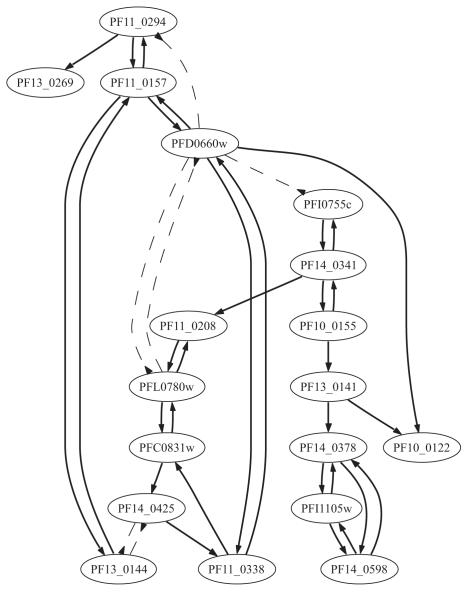
‘OR-NOR’-regulatory interactions of eighteen (18) genes in glycolysis pathway, ‘simultaneous’ gene activities.

**Figure 4:**
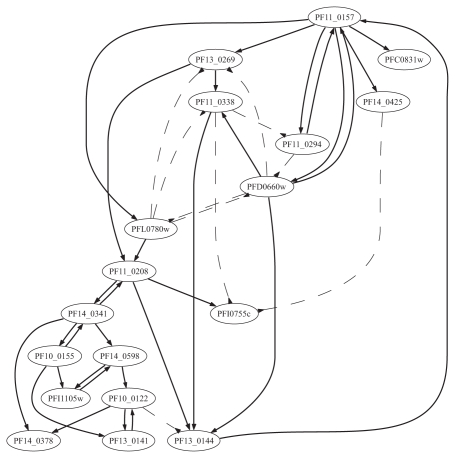
‘OR-NOR’-regulatory interactions of eighteen (18) genes in glycolysis pathway, ‘time-delay’ gene activities.

We applied our approach for learning the ‘OR’-and ‘OR-NOR’-models from data. Two models have different semantics and can bring slightly different results. Learning the “OR”-model identifies only the activators of a gene, i.e. the model “explains” the non-activity of the gene with the failure of its activators. In the “OR-NOR”-model, the non-activity of the regulatee is also “explained” by the activity of its inhibitors. We applied model learning for each gene in the dataset, considering all other genes as candidate regulators. We have considered both ‘simultaneous’ and ‘time delay’ situations. The results of our ‘simultaneous’ and ‘time delay’ learning of the ‘OR-NOR’-model for 18 genes of the glycolysis pathways are summarized in the Tables 2 and 3, and represented graphically in the Figures 3 and 4. The graphs were generated with the program GraphViz (www.graphviz.org). The results of the ‘simultaneous’ and ‘time delay’ learning of the ‘OR’-model are displayed in supplementary Tables 1 and 2 and supplementary Figures 1 and 2.

The ‘OR-NOR’-learning mostly supported the results of the ‘OR’-model but found some more activators and inhibitors with increased accuracy. The regulatory network in Figure 3 reveals the strategic position and hence the key regulatory role of the genes PF11_0157, PFD0660w, PF14_0341 and PF13_0141. The inhibitory connections between the genes PFD0660w and PFL0780w, from the gene PFD0660w to the gene PFI0755c, and between the genes PF14_0425 and PF13_0144 might indicate three groups of genes working in timely separated manner. One group include the genes PF11_0157, PF13_0144, PF11_0294, PF13_ 0269 and PFD0660w. The second group contains the genes PFI0755c, PF14_0341, PF10_ 0155, PF13_0141, PF10_0122, PF14_0378, PFI1105w, PF14_0598, where the last three genes are closely connected with each other. The third group is: PF11_0208, PFL0780w, PFC0831w, PF14_0425 and PF11_0338. Using a query tool titled “Identify Genes based on Predicted Functional Interaction” from PlasmoDB, which is based on the data obtained from the work of Date and Stoeckert(8), the second group functionality or connectivity was overwhelmingly confirmed except for genes PF13 _0141 and PF14_0378. We will suggest that a further biological studies should be carried out to check our prediction here. Furthermore, in group three, using this tool, we are able to show that genes PF11_0338 and PFL0780w, PFL0780w, PF11_0338 and PFC0831w and PFC0831w and PFL0780w are functionally connected. This tools could not verify the functional connectivity of PF11_0208 and PF14_0425 as we have shown theoretically. Based on the correctness of our predictions so far, we will suggest that these and group one connectivities (including their regulatory modalities) as shown here should also be tested biologically.

The genes PFD0660w, PF14_0341, PF11_0338, and PF14_0425 present interesting crosspoints between the separate groups. In the recent review (24) that cataloguizes the various drug targets of P.f., three genes PF14_0341, PF13_0141 and PF14 _0425 which encode three important energy metabolites, namely enzymes EC 5.3.1.9 (glycose-6 phosphate isomerase), EC 1.1.1.27 (lactate dehydrogenase) and EC 4.1.2.13 (aldolase) were stated as possible drug targets genes. Our theoretical finding predicts the important regulatory role of these genes in the glycolysis. The regulatory interactions of these genes to others reconstructed by our learning procedure should be verified in biological studies. Furthermore, biological literature supports our prediction that the gene PF14 _0598 is been activated by PF14_0378 (20).

The ‘time delay’ regulatory network (see Table 3 and Fig. 4) suggests the key regulatory role of the genes PF11_0157, PF11_0208, PF14_0341 and PF10_0155. The graph in Figure 4 also reveals the groups of closely connected genes. Interestingly, the gene PF10_0155 is connected to both enzyme genes PF14_0341 and PF13_0141. It was shown experimentally that the gene PF13_0269 is been activated by PF11_0157(22) as we have predicted here. It is interesting to note that the metabolic pathway maps with enzymes for the P.f glycolysis pathway available at KEGG database supports our predicted interaction depicted in Figure 4. However, Figure 4 contains more informations and therefore can be used to update the KEGG database.

Barrera et al. (1) applied their probabilistic genetic network approach to the gene expression profiles of ten enzymes from the glycolytic pathway of P.f. Note that apart from the target genes that coded for all the 10 enzymes pertaining to the glycolytic pathway, included in their analysis are also 40 best predictors for each glycolytic target (289 distinct oligos in total). The authors assumed the ’time delay’ regulation. Their estimation procedure is based on the conditional entropy minimization to discover subsets of genes predicting the target gene at best. Their results provided a biologically meaningful list of genes with putatively similar functions, which are also obtained in (5), but it is not possible to study the modalities of the genes regulation with their approach. Furthermore, the authors worked on the level of oligonucleotides i.e. representing one gene with multiple oligonucleotides, thus inserting bias from the statistical point of view. Among the oligos for target genes that code for all the 10 enzymes pertaining to the glycolytic pathway, in their network, the oligos for the genes PF14_0341 and PFI0755c are the only ones that shared direct contact. This is also shown to be so in our predictions of (Fig 3) in addition to the modality of their regulation.

Khanin and Wit (13) derived their overall malaria gene network in the intraerythrocytic development cycle based on the 3048 genes. Note that Khanin and Wit (13) averaged the values from multiple oligonucleotides representing same gene. They claim that nine genes of the glycolysis pathway share five links among themselves and show that the probability of nine randomly picked genes having 5 links is 0.01% given the connectivity matrix. Apart from the fact that, their output provided no hints on the altitude of the genes during regulation, we found out that theses genes share more than five links.

### 3.2 Plastid genome

We considered the set of genes annotated to make up the plastid genome, otherwise known as the apicoplast. We harvested the oligonucleotides representing each of the 26 putative apicoplast genomeencoded proteins listed from the DeRisi’s laboratory (malaria.ucsf.edu). In this case, we found in the 3D7 gene expression dataset (S01_ 3D7_Raw.txt), taking also from the DeRisi malaria site, that only one oligonucleotide was used to represent each putative protein encoded genes of the plastid genome.

The apicoplast can be thought as a cell living within another cell (here P.f) and contains all the familiar cellular processes such as DNA replication, transcription, translation, fatty acid synthesis, isopentenyl diphosphate synthesis, aromatic amino acid synthesis and the heme synthesis (19).

**Figure 5:**
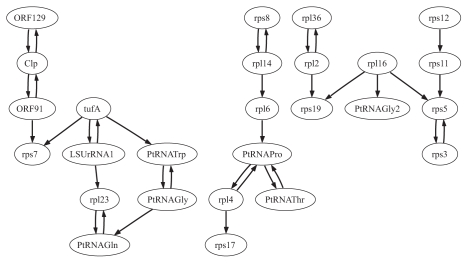
‘OR-NOR’-regulatory interactions of twenty six (26) genes in the plastid genome, ‘simultaneous’ gene activities.

**Figure 6:**
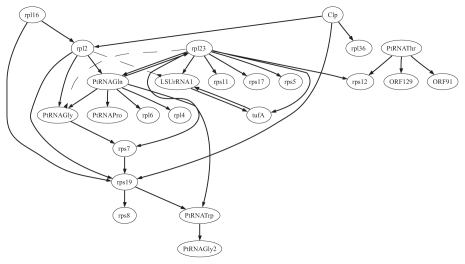
‘OR-NOR’-regulatory interactions of twenty six (26) genes in the plastid genome, ‘time delay’ gene activities.

The results of our ‘simultaneous’ and ’time delay’ learning of the ‘OR-NOR’-model for 26 genes of the apicoplast are summarized in the Tables 5 and 6, and represented graphically in the Figures 5 and 6. The results of the ’simultaneous’ and ’time delay’ learning of the ‘OR’-model are displayed in Supplementary Tables 3 and 4 and Supplementary Figures 3 and 4. The graphs for ‘simultaneous’ ‘OR’-and ‘OR-NOR’-regulation contain disconnected components suggesting the groups of closely related genes. One can see the strategical positioning of the genes ORF129, Clp, ORF91, tufA, PtRNA-Pro, and rpl16 indicating the key regulatory role of these genes. We discovered from the literature that the genes rpl23, tufA and rpl16 has been tested in wet experiments and have been marked as putative drug/herbicide targets (19). The observation of the ‘time delay’ regulatory interactions inferred further suggest the important role of Clp, rpl23, rpl16 but also of the genes rpl2, PtRNAGln and PtRNAThr. The gene rps19 was inferred to be regulated by many other genes. The graph of the ‘time delay’ ‘OR-NOR’-regulation is much more connected than that of the ’simultaneous’.

Table 4 shows the functional interconnection as derived in (Fig. 2) (B) of Barrera et al. (1). There are a lot of deviations, contactwise, in the contacts we predicted. Using the genes rpl23, tufA and rpl16 that has been marked as putative drug/herbicide targets in Ralph et al. (19), we noted that our derived networks in Figures 5;6 and Figures 3;4 of the supplementary material suggested the correctness of Ralph et al. predictions on genes rpl23, tufA and rpl16 than that of Barrera et al.

From all the information that we could gathered from PlasmoDB, DeRisi malaria site, KEGG database and the metabolic maps of the apicoplast from the work of Barrera et al. which we have encapsulated in Table 4, presently, nothing else is known about the metabolic maps of the apicoplast genes. So our work provides the first insight into the apicoplast genes regulatory connections.

## 4 Conclusion

In the present work we have elucidated regulatory interactions of the genes of P.f. in glycolysis pathway and apicoplast metabolism. Some of our predictions found support in the biological literature, others would be worthwhile to verify in the ‘wet’ laboratory. We have proposed the groups of closely connected genes (pathways) and crosspoints between them.

Our approach is based on the Bayesian learning of the probabilistic graphical model explicitly representing the logic dependencies between a gene and its regulators. Our method allows for elucidating more complex multigene relations which go beyond pairwise relations retrieved by other approaches. Also, we derive sparse connections between the genes, which can be further interpreted and validated in the laboratory, as opposed to the previous authors (e.g. Khanin and Wit, 2004). An important advantage of the Bayesian approach is that it enables the inclusion of “subjective” prior information into the model. In this study we used the subjective prior specification to enforce the number of gene regulators to lie in the small range. Potentially, one could define priors aiming to incorporate previous biological knowledge into the model learning.

We have identified a few genes (like PF11_0157, rpl23) which regulate many other genes (master activators). Further theoretical extension of our work would be to collect potentially commonly regulated genes, scanning their upstream regions for commonly present, conserved sequence motifs (for example, by means of the PhyME algorithm (21)), computing a probability weight matrix from such motifs and scanning the genome of P.f. for further genes that harbor these motifs in their upstream region. Our initial work in this workflow revealed a set of three motifs commonly contained in seven genes (PFL0780w, PF14_0425, PF13_ 0269, PF11_0294, PF13_0144, PFC0831w and PFD0660w) computationally predicted to be activated by PF11_0157 (Tachado et al. (22) has shown experimentally that PF13_0269 is been activated by PF11_0157). Restricting to high confidence presence of the motif, and requiring at least two of the three motifs to be present, a set of seven genes (PFL1160c, PFB0480w, PFI0260c, PFI1605w, MAL8P1.143, PF14_0363 and PF14_0472) were found that show remarkable correlation of their expression values with those of the glycolysis genes used to compile the motifs.

## Supplementary Material

S. Figure 1 ‘OR’-regulatory interactions of eighteen (18) genes in the glycolysis pathway, ’simultaneous’ gene activities.

S. Figure 2 ‘OR’-regulatory interactions of eighteen (18) genes in the glycolysis pathway, ’time delay’ gene activities.

S. Figure 3 ‘OR’-regulatory interactions of the twenty-six (26) genes in the plastid genome,’simultaneous’ gene activities.

S. Figure 4 ‘OR’-regulatory interactions of twenty six (26) genes in the plastid genome, ‘time delay’ gene activities.

S. Table 1 ‘OR’-regulatory interactions of eighteen (18) genes in the glycolysis pathway, ’simultaneous’ gene activities.

**Table:** 

Genes	Activators	Accuracy (%)
PFD0660w	PF11_0157, PF11_0338	0.83
PFI0755C	PF14_0341	0.92
PFL0780w	F11 0208	0.79
PFC0831w	no	no
PF10_0155	PF14_0341	0.98
PF10_0122	PFD0660w, PF13_0141	0.79
PFI1105w	no	no
PF11_0157	PF11_0294, PFD0660w, PF13_0144	0.83
PF11_0294	PF11_0157	0.62
PF11_0338	PFD0660w	0.83
PF11_0208	F14_0341, PFL0780w	0.94
PF13_0141	no	no
PF13_0269	PF11_0294	0.89
PF13_0144	PF11_0157	0.57
PF14_0425	no	no
PF14_0598	F14_0378	0.94
PF14_0341	PF10_0155, PFI0755c	1
PF14_0378	PF13_0141, PF14_0598	0.98

S. Table 2 ‘OR’-regulatory interactions of eighteen (18) genes in the glycolysis pathway, ’time delay’ gene activities

**Table:** 

Genes	Activators	Accuracy (%)
PFD0660w	PF11_0338, PF11_0157	0.83
PFI0755C	PF11_0208	0.87
PFL0780w	PF11_0157	0.65
PFC0831w	PF11_0157	0.83
PF10_0155	PF14_0341	0.98
PF10_0122	no	no
PFI1105w	no	no
PF11_0157	PF13_0144, PF11_0294, PFD0660w	0.85
PF11_0294	PF11_0157	0.63
PF11_0338	PF13_0269, PFD0660w	0.83
PF11_0208	PFL0780w, PF13_0269, PF14_0341	0.96
PF13_0141	PF10_0122	0.85
PF13_0269	PF11_0157	0.90
PF13_0144	PF11_0157	0.58
PF14_0425	PF11_0157	0.87
PF14_0598	PF14_0341, PFI1105w	0.94
PF14_0341	PF11_0208	0.94
PF14_0378	PF14_0341, PF10_0122	0.90

S. Table 3 ‘OR’-regulatory interactions of twenty six (26) genes in the plastid genome, ‘simultaneous’ gene activities.

**Table:** 

Genes	Activators	Accuracy (%)
Clp	ORF91	0.94
LSUrRNA1	PtRNAGly2, tufA	0.68
ORF91	Clp	0.94
ORF129	Clp	0.89
rp12	rp136	0.83
rp14	no	no
rp16	rp114	0.94
rp114	rps8	0.96
rp116	no	no
rp123	LSUrRNA1, PtRNAG1n	0.68
rp136	rp12	0.83
rps3	rps5	0.91
rps5	rp116, rps3	0.94
rps7	ORF91, tufA	0.92
rps8	rp114	0.96
rps11	rps12	
rps12	no	no
rps17	no	no
rps19	rp116, rp12	0.89
PtRNAG1n	rp123	0.68
PtRNAG1y	PtRNATrp	0.87
PtRNAGly2	no	no
PtRNAPro	rp14, PtRNAThr	0.98
PtRNAThr	no	no
PtRNATrp	tufA	0.87
tufA	LSUrRNA1	0.6

S. Table 4 ‘OR’-regulatory interactions of twenty six (26) genes in the plastid genome, ‘time delay’ gene activities.

**Table:** 

Genes	Activators	Accuracy (%)
Clp	no	no
LSUrRNA1	rpl23	0.56
ORF91	no	no
ORF129	no	no
rpl2	no	no
rpl4	no	no
rpl6	no	no
rpl14	no	no
rpl16	no	no
rpl23	no	no
rpl36	Clp	0.87
rps3	no	no
rps5	rpl23	0.73
rps7	rpl23, PtRNAGly	0.62
rps8	rps19	0.79
rps11	rpl23	0.75
rps12	rpl23	0.63
rps17	rpl23	0.75
rps19	rps7	0.75
PtRNAGln	rpl23	0.67
PtRNAGly	no	no
PtRNAGly2	no	no
PtRNAPro	no	no
PtRNAThr	no	no
PtRNATrp	PtRNAGln, rps19	0.81
tufA	LSUrRNA1, rpl23	0.65

## Figures and Tables

**Table 1:** Hypothetical functions and EC numbers for genes of the glycolysis pathway.

Gene	Product Description	EC Numbers
PF10_0122	phosphoglucomutase, putative	5.4.2.2
PF10_0155	enolase	4.2.1.11
PF11_0157	glycerol-3-phosphate dehydrogenase, putative	1.1.1.8
PF11_0208	phosphoglycerate mutase, putative	5.4.2.1
PF11_0294	ATP-dependent phosphofructokinase, putative	2.7.1.11; 2.7.1.90
PF11_0338	Aquaglyceroporin	
PFL0780w	glycerol-3-phosphate dehydrogenase, putative	1.1.1.8
PF13_0141	L-lactate dehydrogenase	1.1.1.27
PF13_0144	oxidoreductase, putative	1.1.1.-; 1.1.1.27
PF13_0269	glycerol kinase, putative	2.7.1.30
PF14_0341	glucose-6-phosphate isomerase	5.3.1.9
PF14_0378	triose-phosphate isomerase	5.3.1.1
PF14_0425	fructose-bisphosphate aldolase	4.1.2.13
PF14_0598	glyceraldehyde-3-phosphate dehydrogenase	1.2.1.12
PFC0831w	triosephophate isomerase, putative	5.3.1.1
PFD0660w	phosphoglycerate mutase, putative	5.4.2.1
PFF1155w	hexokinase	2.7.1.1
PFF1300w	pyruvate kinase, putative	2.7.1.40
PFI0755c	6-phosphofructokinase, putative	2.7.1.11
PFI1105w	Phosphoglycerate kinase	2.7.2.3

**Table 2:** ‘OR-NOR’-regulatory interactions of eighteen (18) genes in the glycolysis pathway, ‘simultaneous’ gene activities.

Genes	Activators	Inhibitors	Accuracy (%)
PFD0660w	PF11_0157, PF11_0338	PFL0780w	0.91
PFI0755C	PF14_0341	PFD0660w	0.94
PFL0780w	PF11_0208, PFC0831w	PFD0660w	0.92
PFC0831w	PFL0780w	no	0.81
PF10_0155	PF14_0341	no	0.98
PF10_0122	PFD0660w, PF13_0141	no	0.85
PFI1105w	PF14_0598	no	0.94
PF11_0157	PF11_0294, PFD0660w, PF13_0144	no	0.83
PF11_0294	PF11_0157	PFD0660w	0.77
PF11_0338	PFD0660w, PF14_0425	no	0.83
PF11_0208	PF14_0341, PFL0780w	no	0.94
PF13_0141	PF10_0155	no	0.91
PF13_0269	PF11_0294	no	0.89
PF13_0144	PF11_0157	PF14_0425	0.62
PF14_0425	PFC0831w	PF13_0144	0.89
PF14_0598	PF14_0378, PFI1105w	no	0.94
PF14_0341	PF10_0155, PFI0755c	no	1
PF14_0378	PF13_0141, PF14_0598	no	1

**Table 3:** ‘OR-NOR’-regulatory interactions of eighteen (18) genes in glycolysis pathway, ’time delay’ gene activities

Genes	Activators	Inhibitors	Accuracy(%)
PFD0660w	PF11_0157	PFL0780w, PF11_0294	0.88
PFI0755C	PF11_0208	PF14 0425, PF11_0338	0.85
PFL0780w	PF11_0157	PFD0660w	0.71
PFC0831w	PF11_0157	no	0.83
PF10_0155	PF14_0341	no	0.98
PF10_0122	PF14_0598, PF13_0141	no	0.81
PFI1105w	PF14_0598, PF10_0155	no	0.96
PF11_0157	PF11_0294, PFD0660w, PF13_0144	no	0.85
PF11_0294	PF11_0157	PF11_0338	0.67
PF11_0338	PF13_0269, PFD0660w	PFL0780w	0.81
PF11_0208	PF13_0269, PFL0780w, PF14_0341	no	0.96
PF13_0141	PF10_0122, PF10_0155	no	0.90
PF13_0269	PF11_0157	PFL0780w, PFD0660w	0.90
PF13_0144	PF11_0338, PF11_0208, PFD0660w	PF10_0122	0.69
PF14_0425	PF11_0157	no	0.87
PF14_0598	PF14_0341, PFI1105w	no	0.94
PF14_0341	PF10_0155, PF11_0208	no	0.96
PF14_0378	PF10_0122, PF14_0341	no	0.90

**Table 4:** Barrera et al.(1) predicted pairwise interactions of the twenty six(26) genes in the plastid genome.

Contact	First gene	Other genes involved
1	**rpl23**	PtRNAPro
2	PtRNAPro	rpl23
3	rps12	rps19, tufA
4	ORF129	rpl2, rps5, rps7, ORF91
5	rpl2	rps19, rps5
6	rps17	rpl2
7	rps3	rpl2
8	rps19	tufA, rps12, Clp, rps5, rps5, rpl14, rpl16, rpl38
9	rpl2	rps11, ORF129, rps12, ORF91, rpl14, ORF91
10	**rpl16**	rps19
11	rpl36	rpl16, rps19
12	**tufA**	rpl36, rps11, rps12
13	ORF91	ORF129, rpl2
14	rpl14	rpl2, rps19
15	rps5	rps19, rpl2, Clp
16	Clp	rpl2, rps19, rps5
17	rps7	rps5
18	PtRNAPHE	ORF129

**Table 5:** ‘OR-NOR’-regulatory interactions of twenty six (26) genes in the plastid genome, ‘simultaneous’ gene activities.

Genes	Activators	Inhibitors	Accuracy(%)
Clp	ORF129, ORF91	no	0.94
LSUrRNA1	tufA	no	0.60
ORF91	Clp	no	0.94
ORF129	Clp	no	0.89
rpl2	rpl36	no	0.83
rpl4	PtRNAPro	no	0.96
rpl6	rpl14	no	0.94
rpl14	rps8	no	0.96
rpl16	no	no	no
rpl23	LSUrRNA1, PtRNAGln	no	0.68
rpl36	rpl2	no	0.83
rps3	rps5	no	0.91
rps5	rpl16, rps11, rps3	no	0.94
rps7	ORF91, tufA	no	0.92
rps8	rpl14	no	0.96
rps11	rps12	no	0.87
rps12	no	no	no
rps17	rpl4	no	0.92
rps19	rpl16, rpl2	no	0.89
PtRNAGln	rpl23, PtRNAGly	no	0.85
PtRNAGly	PtRNATrp	no	0.87
PtRNAGly2	rpl16	no	0.75
PtRNAPro	rpl4, rpl6, PtRNAThr	no	1
PtRNAThr	PtRNAPro	no	0.83
PtRNATrp	PtRNAGly, tufA	no	0.87
tufA	LSUrRNA1	no	0.60

**Table 6:** ‘OR-NOR’-regulatory interactions of twenty six (26) genes in the plastid genome, ’time delay’ gene activities.

Genes	Activators	Inhibitors	Accuracy(%)
Clp	no	no	no
LSUrRNA1	rpl23, tufA	rpl2	0.60
ORF91	PtRNAThr	no	0.85
ORF129	PtRNAThr	no	0.83
rpl2	Clp, rpl16	no	0.83
rpl4	PtRNAGln	no	0.81
rpl6	PtRNAGln	no	0.77
rpl14	no	no	no
rpl16	no	no	no
rpl23	PtRNAGln	no	0.67
rpl36	Clp	no	0.87
rps3	no	no	no
rps5	rpl23	no	0.73
rps7	rpl23, PtRNAGly	no	0.75
rps8	rps19	no	0.79
rps11	rpl23	no	0.75
rps12	rpl23, PtRNAThr	no	0.84
rps17	rpl23	no	0.75
rps19	Clp, rpl16, rpl2, rps7	no	0.83
PtRNAGln	rpl2, rpl23	no	0.81
PtRNAGly	rpl2, PtRNAGln	rpl23	0.81
PtRNAGly2	PtRNATrp	no	0.65
PtRNAPro	PtRNAGln	no	0.85
PtRNAThr	no	no	no
PtRNATrp	rps19, PtRNAGln	no	0.81
tufA	LSUrRNA1, rpl23	no	0.65
