# Supplementary figures and images for: New Insights into the Genetic Regulation of Plasmodium Falciparum Obtained by Bayesian Modeling
Source: Gene Regul Syst Bio. 2007 Nov 29;1:137–49. (PMC2759121)

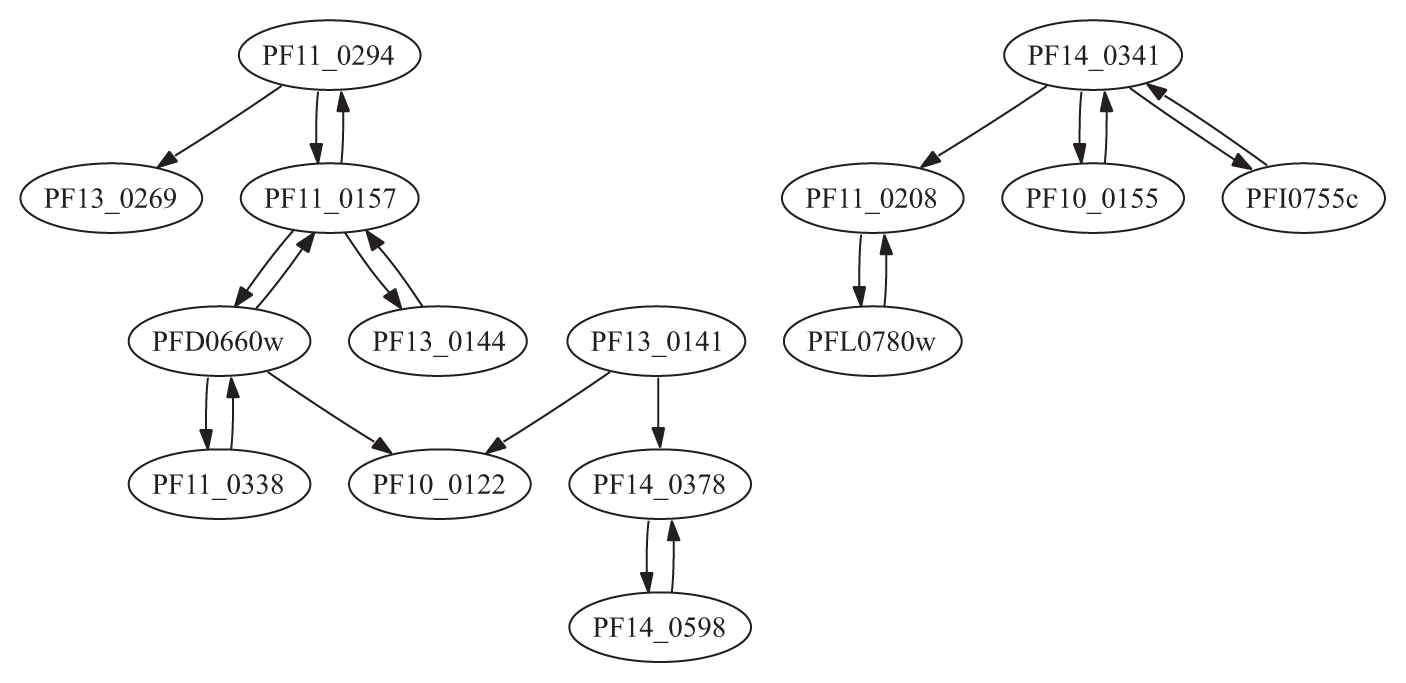

Supplement: S. Figure 1 — ‘OR’-regulatory interactions of eighteen (18) genes in the glycolysis pathway, ’simultaneous’ gene activities. [file grsb-2007-137f7.tif]

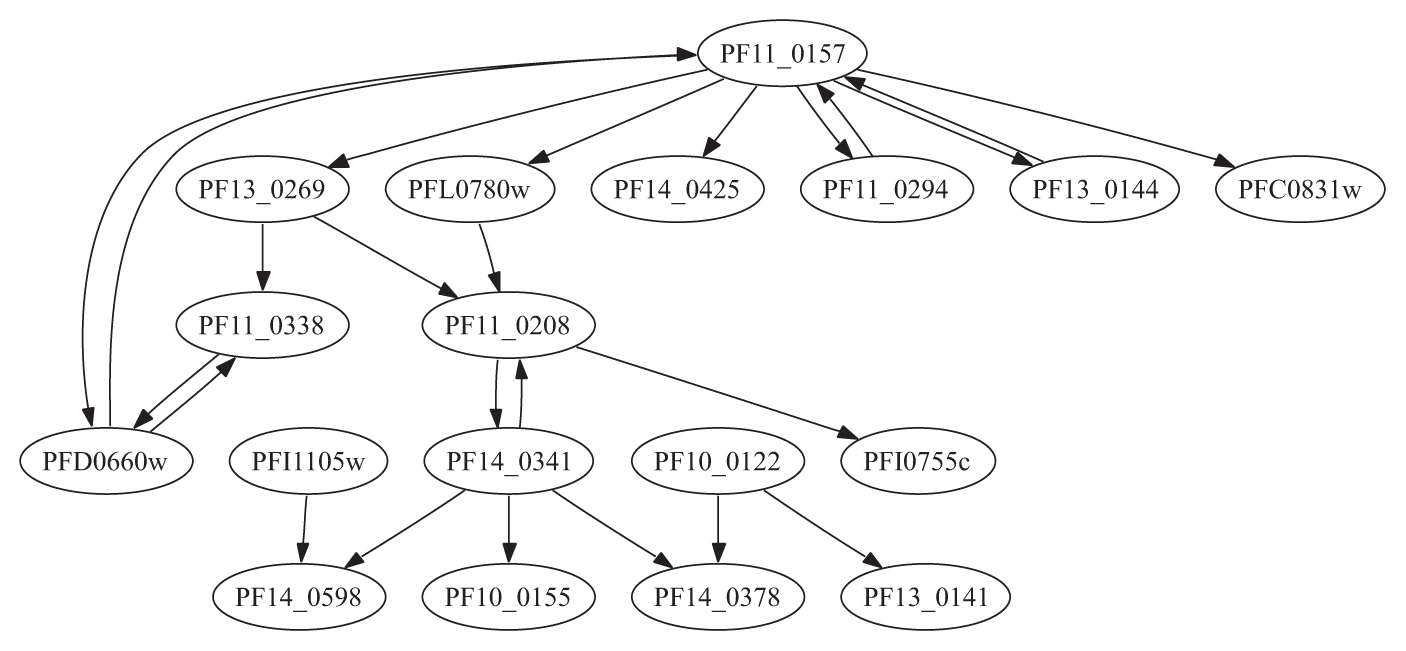

Supplement: S. Figure 2 — ‘OR’-regulatory interactions of eighteen (18) genes in the glycolysis pathway, ’time delay’ gene activities. [file grsb-2007-137f8.tif]

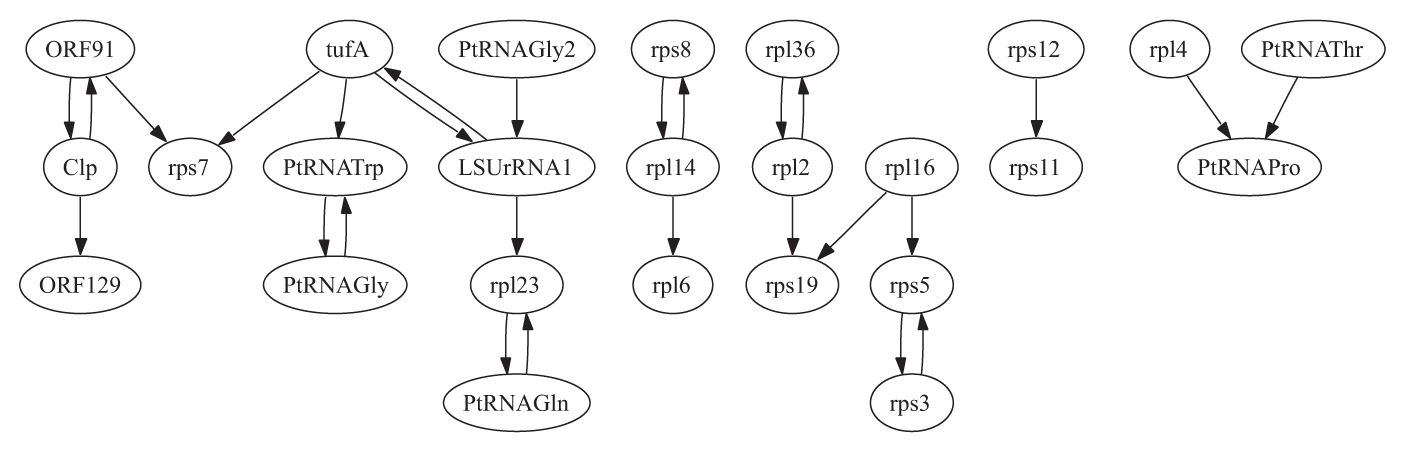

Supplement: S. Figure 3 — ‘OR’-regulatory interactions of the twenty-six (26) genes in the plastid genome,’simultaneous’ gene activities. [file grsb-2007-137f9.tif]

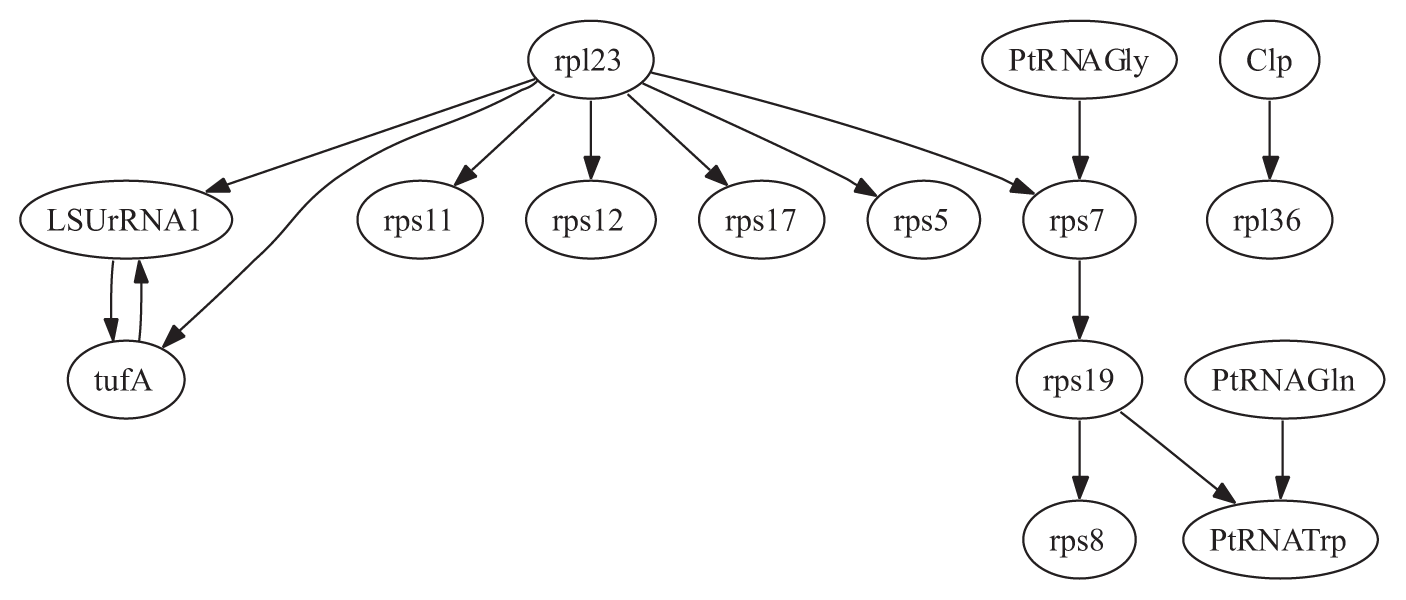

Supplement: S. Figure 4 — ‘OR’-regulatory interactions of twenty six (26) genes in the plastid genome, ‘time delay’ gene activities. [file grsb-2007-137f10.tif]
